# A Review of Corneal Blindness: Causes and Management

**DOI:** 10.7759/cureus.30097

**Published:** 2022-10-09

**Authors:** Shivangi C Tidke, Pravin Tidake

**Affiliations:** 1 Ophthalmology, Jawaharlal Nehru Medical College, Datta Meghe Institute of Medical Sciences, Wardha, IND

**Keywords:** prevention, eye banking, cornea, lamellar keratoplasty, bioengineering

## Abstract

Corneal blindness refers to a group of eye disorders that change the corneal transparency, causing corneal scarring and blindness. The leading causes of corneal blindness include infectious causes, i.e., due to bacteria, viruses, fungi, and protozoa. The most common predisposing factors are trauma, contact lens usage, or the use of steroid medications. The various other diseases included are trachoma, dry eye disease, keratoconus, ophthalmia neonatorum, and non-infectious uveitis.

Various clinical modalities are used for treating corneal blindness, including organ transplantation. Organ donation is cumbersome as various ethical and other factors are involved. Hence the concept of eye banking was introduced to meet the increasing demand for donors of the cornea. The eye bank's role is harvesting, processing, and keeping a record of the cornea being transplanted and donated. Furthermore, various recent advancements have been made for lamellar keratoplasty surgeries, including bioengineered corneas to fulfil the need for the unavailability of donors for the cornea. Various specific health interventions have been implemented to reduce the prevalence of corneal blindness globally.

For proper management of corneal blindness, we have three components that are needed to be taken care of: prevention of corneal blindness, appropriate treatment modalities, and providing adequate rehabilitation services to the patients. This review encompasses the main reasons for corneal blindness and the management and treatment modalities available for the patients.

The terms cornea, corneal blindness, treatment, management, causes, and complications were used for the review article on PubMed.

## Introduction and background

Although most of the eye's focusing power comes from the cornea, the focus is fixed. By altering the lens' shape, accommodation, i.e., the focussing of light to provide a clearer vision of close objects, can be achieved. The epidemiology of cornea is too extensive, including viral ocular disorders, which lead to corneal scarring and inflammatory conditions and finally lead to functional blindness [1]. Globally, the foremost reasons for blindness are uncorrected refractive error, glaucoma, and diabetic retinopathy. With increasing age, the number of people affected by vision loss also increases [2]. To cope with the rising cases, we should take the initiative to set up large-scale eye camps to address these patients. The government should develop various public health programmes and awareness programmes to deal with the current havoc created due to loss of vision targeting the senior population and neonates and children. Approximately 1.4 million children have blindness globally, meaning they are more likely to be in a lower socio-economic class and suffer from socio-economic deprivation [3]. 

## Review

Anatomy of cornea

When the cornea is touched, an involuntary reaction to close the eyelid occurs because the cornea possesses unmyelinated nerve endings that are subtle to feel, temperature, and chemicals. A healthy cornea has no need for or necessity for blood vessels since transparency is of utmost significance. The anterior-most part of the eye is the transparent structure forming the anterior one-third of the outer layer called the cornea [4]. 

The cornea comprises six layers: the corneal endothelium, Dua's layer, Bowman's membrane, corneal stroma, and corneal epithelium. The thickness of the cornea in an adult is 550 microns [4]. The main functions of the cornea are the following: to protect structures inside the eye, the structural barrier, and against environmental infections, and to contribute to the eye’s two-third refractive power [5].

It is constituted of two components: the cellular component and the acellular component. Collagen and glycosaminoglycans are included in acellular details, whereas endothelial cells, keratocytes and epithelial cells are included in the cellular part [4]. 

Causes of corneal blindness

**Figure 1:**
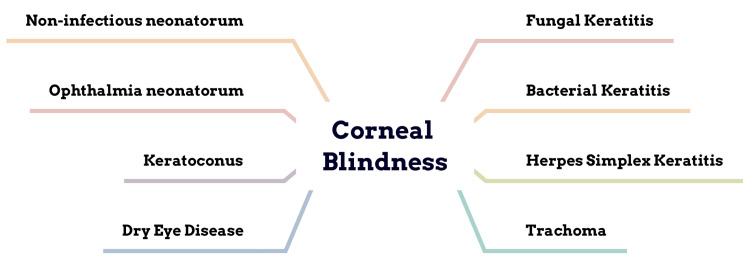
Causes of corneal blindness Image credit: Author Shivangi C. Tidke

The various causes leading to corneal blindness are depicted in Figure 1.

Bacterial Keratitis

Bacterial keratitis is the most common type of infectious keratitis in most countries worldwide. The most common bacteria responsible for causing bacterial keratitis is coagulase-negative staphylococci followed by *Staphylococcus aureus, Streptococci spp., Pseudomonas aeruginosa *and* Enterobacteriaceae spp* [6].

Fungal Keratitis

There are various species of fungi that cause fungal keratitis eventually leading to corneal blindness. The most common fungi that are involved are *Candida, Aspergillus, *and *Fusarium* [7]. In India, the most commonly involved species is *Aspergillus* which is then followed by *Fusarium* [8,9]. The prevalence of specific species of fungi depends upon various specific risk factors such as temperature, climatic conditions, and urbanization of that region. The patient-specific risk factors include trauma and contact lens use [10].

The pathophysiology behind fungal keratitis is a defect in the corneal epithelium which gives entry to the corneal stroma. This is the reason for the increased prevalence of this disease in patients with trauma [11].

Herpes Simplex Keratitis (HSK)

The infection caused by the herpes simplex virus is another cause which progressively leads to blindness due to corneal involvement. Keratitis instigated by herpes simplex virus (HSV) type 1 is a primary reason for contagious blindness. Epithelial or dendritic keratitis is the most typical manifestation. With recurrent illness, herpes stromal keratitis can cause progressive corneal opacification and visual loss [12].

The mouth, genitalia, and eyes, however, are the most typical sites of infection in a person with a healthy immune system. Very young toddlers and, in rare cases, adults may also get brain infections. In affluent nations, HSV eye illnesses are the principal infectious reason for corneal blindness [13]. The three types of stromal keratitis caused by HSV are endothelial subtype, epithelial subtype, and stromal subtype. In disciform HSK, the DNA value of the HSV is reduced as compared to the dendritic HSK. The laboratory boosts confidence in identifying HSK subdivisions with the combination of the HSV Immunoglobulin A by the enzyme-linked immunosorbent assay (ELISA) by the use of tear samples along with the procedure of real-time polymerase chain reaction (PCR) [14].

Trachoma

Currently, trachoma is the leading cause of preventable blindness globally. Chlamydia trachomatis bacterial infection is spread through sexual contact, causing chlamydia. It is the furthermost often stated microbial disease in the United States. It is the most prevalent sexually transmitted disease (STD) in the entire world. It produces trachoma, the most pervasive infectious factor behind blindness worldwide, an eye infection [15].

Dry Eye Disease

It was previously known as Keratoconjunctivitis sicca, which was given by Henrik Sjogren. He also established the triad, which included joint pain, dryness of mouth and Keratoconjunctivitis sicca [16]. The patient presents with the symptoms of foreign body existence, itchy and gritty eyes accompanied by excess tears and blurred vision. Furthermore, when the disease is not treated, it leads to further worsening of conditions causing discomfort and eventually causing blindness. The most common affected population is the senior population; the disease prevalence is seen more in females as compared to males. The various causes of dry eye disease are ocular surface dysfunction, blink rate, autoimmune diseases and other disorders [17]. The risk factors that are mainly responsible for the condition are the female gender and the advancement of age. The hormonal imbalance in the females further aggravates the symptoms like the decrease of tear production significantly around 60 years, along with the effect on meibomian gland function and the density of goblet cells of the conjunctiva [16].

The assessment and diagnosis of the diseases are based upon the questionnaires that are specifically developed for dry eye diseases like symptom assessment in dry eye (SANDE) and ocular comfort index (OCI) [18].

Keratoconus

Keratoconus is an illness that causes thinning of the cornea, eventually causing reduced visual acuity and irregular astigmatism. It is a bilaterally asymmetrical disease associated with ocular inflammation. The various risk factors associated with keratoconus are allergy, atopy, eye rubbing and various environmental and familial factors, which are mediated by immunoglobulin E (IgE) [19].

Ophthalmia Neonatorum

This is the conjunctiva disease, a kind of conjunctivitis observed in neonates. This disease frequently spreads following a vaginal birth and is associated with severe side effects such as ocular perforation and ulceration, which may result in lifelong blindness. According to research, this eye condition is caused by a number of microbes, including Chlamydia trachomatis, N. gonorrhoea, infection of the virus, and bacteria from the skin and intestinal system [20].

Non-infectious Neonatorum

It is an autoimmune disease, which is one of the leading reasons for blindness that can be prevented by various measures.

Management and treatment of corneal blindness

For evaluation of the infectious keratitis, the primary step is the sample collection for culture and direct microscopy using various stains. There might be a need for corneal biopsy for deeper infections [11]. The gold standard techniques for diagnosing the infectious cause leading to corneal blindness remain the same, that is, Gram staining and culture methods which give the results instantaneously [21]. For confirmation of the diagnosis, a PCR is used occasionally because of its high sensitivity.

Fungal Keratitis

The medical treatment currently available includes antifungal agents which are fungistatic that increase the duration of treatment for complete eradication of the causative agent. The drug of choice is topical natamycin 5% and the other topical antifungal agents that can be given are amphotericin B, which is used specifically for yeasts; voriconazole; and itraconazole.

For the patients who do not respond to the given medical treatment, surgical interventions have been used for their treatment. The interventions include lamellar keratoplasty and therapeutic keratoplasty [11].

Bacterial Keratitis

The treatment of choice for bacterial keratitis is the use of topical antibiotics. Fluoroquinolones are most commonly used. Even anti-collagenases and steroids are used for the treatment [22].

In the case of bacterial keratitis, topical antibiotics are the most preferred and primary stay treatment regimen. Even anti-collagenases and steroids can be used in bacterial keratitis [22]. Voriconazole is used for fungal keratitis, which is a newer generation triazole because of its tremendous ocular penetration [23]. The ultimate target for managing the above condition is to decrease inflammation and avoid further eye complications [24].

Dry Eye Disease

Questionnaires like Fluorescin break up time or Ocular Surface Disease Index can be used for diagnosing dry eye disease along with Schirmer's test for detecting decreased tear production; the assessment can also be carried out by stains and by cytology to find out the ocular damage [25,26].

Dry eye disease management can be done by keeping the ocular environment in control, such as avoiding prolonged exposure to digital devices, avoiding the dry atmosphere and using external protection like contact lenses such as silicon hydrogel and scleral lenses [26]. Even lipids can be used with velocity enhancers for the effective treatment of dry eye disease (DED) [27].

Keratoconus

The early detection of keratoconus can be done by more frequent monitoring of the disease progression and performing the indicated interventions at the time, leading to improved patient outcomes so that the use of transplantation of cornea is reduced to a significant amount [19]. The advances for the diagnosis are the corneal biomechanics, various biomarkers like tear inflammatory cytokine or levels of matrix metalloproteinases in the tear immunoglobulin A or by using artificial intelligence [28].

Various treatment modalities are available for the management of keratoconus and for preventing corneal blindness. To prevent further disease progression, the mainstay clinical modality is corneal cross-linking. Various strategies and new molecules have been implicated in the scleral cross-linking. One of the more unknown molecules isolated from the Streptoverticillium sp., named transglutaminase, does not need photoactivation, which makes the cornea stiffer without causing much damage to the underlying layers of the cornea. The others include numerous keratoplasty procedures, transplantation of the Bowman's layer, additive keratoplasty, and cellular therapies [28]. Keratoconus causes the fragmentation of the Bowman’s layer in the earliest phase of the disease [29].

The biomechanical support to the cornea is provided by the Bowman's layer, which is also responsible for the maintenance of its shape and keeping it sturdy. Hence, if we replace this tissue, we can stop the further progression of the keratoconus to its later stages and prevent blindness [30]. A femtosecond laser was employed to produce the stromal compartment, which decreased the risk of micro-perforation during manual dissection. Recently, intraoperative optical coherence tomography has allowed for better visibility of the dissection plane [31]. The graft is localised at the mid-stromal level in the traditional approach; however, a recent variation describes inserting the graft as an onlay in the subepithelial region [32]. The additive keratoplasty helps to increase the thickness of the cornea along with biomechanical stability. To avoid immune rejection, an approach towards tissue engineering has been preferred, which enabled better outcomes in the case of visual acuity and in terms of biomechanical effects [28]. Diagnosis of non-infectious uveitis is based on clinical symptoms and the association with systemic diseases [33].

Corneal transplantation

One of the treatment modalities that is used for treating corneal blindness is organ donation. The concept of corneal transplantation for the treatment of blindness was first stated by Himly in the year 1813, but the first transplantation surgery was actually performed by Von Hippel in the year 1886 by replacing the cornea of a rabbit [34]. Anterior corneal opacities were initially treated using lamellar keratoplasty, including the selective removal of layers of the cornea. This treatment modality was actually used to treat the disease keratoconus and also the scarring of the cornea, but it was halted since it didn't provide the best visual gain. This might have been because of the imperfect interface or any remaining opacities [35].

The various types of lamellar keratoplasty surgeries that can be done are anterior lamellar keratoplasty (ALK), superficial anterior lamellar keratoplasty (SALK), automated lamellar therapeutic keratoplasty (ALTK) and others.

Immune Privilege

The three barriers that contribute to the ocular immune privilege of the cornea are anatomical, cellular and molecular. The mentioned mechanisms facilitate immune tolerance to donor antigen. The predisposing factors destroying and interrupting the immune privilege are the following: the previous rejection of the graft, vascularized corneal tissue, and ocular inflammation.

When the immune privilege is interrupted, it leads to corneal graft rejection. It is predominantly a cell-mediated pathway [36].

Recent Advancements

Intra-operative Optical Coherence Tomography (iOCT)

Continuous feedback on intraoperative surgical manoeuvres is provided by the iOCT. In Lamellar keratoplasty programmes like superficial anterior lamellar keratoplasty, automated lamellar therapeutic keratoplasty, deep anterior lamellar keratoplasty, Descemet stripping endothelial keratoplasty, and Descemet membrane endothelial keratoplasty, it is beneficial. The centre corneal thickness (CCT) of the donor and host corneas, both of which are significant criteria for choosing the blade size to be utilised in the microkeratome for dissection, may be measured using the iOCT. Additionally, it serves as an intraoperative directing aid when donor tissue is manually prepared so that the issues related to this will be further reduced. With the use of iOCT, appropriate coherence may be carried out in situations of superficial anterior lamellar keratoplasty and automated lamellar therapeutic keratoplasty. The iOCT directs every surgical step in the deep anterior lamellar keratoplasty (DALK) process, from the depth of trephination through graft-host apposition [37].

Femtosecond Laser-Assisted Lamellar Keratoplasty (FALK)

The femtosecond laser can be used for laser and total thickness penetrating keratoplasty (PKP). This keratoplasty has various improvements when compared with the manual one [38].

Bioengineered Cornea

This technique was developed for visual rehabilitation and for managing the gap in the availability of donors. Bioengineered cornea includes replacing the part of the cornea or the whole of the diseased cornea [39]. These include various methods ranging from the use of keratoprosthesis that actually supersedes the function of the cornea to the most recent advancement of tissue-engineered hydrogels, which assist in regenerating the tissue of the host [40].

Prevention

Various public health interventions are needed to decrease the prevalence of corneal blindness. Measures like vitamin A supplements and advice regarding the modification of nutrition, i.e., nutritional assessment and the vaccination against measles can prevent xerophthalmia, which is caused due to Vitamin A deficiency. Implementing the SAFE strategy, which includes Surgery for trichiasis, Antibiotics for infection, Facial cleanliness and Environmental improvement to control transmission, can successfully prevent trachoma, which is caused by Chlamydia trachomatis, causing corneal opacification leading to corneal blindness and decreasing its prevalence. Onchocerciasis can cause blindness due to inflammation caused by the Onchocerca volvulus, which can be controlled by the ivermectin distribution in public along with the control of the Simulium fly. In less developed and developing countries, traumatic corneal abrasion is the most common precipitating factor for blindness due to corneal involvement. Hence, to prevent such blindness due to trauma, a prophylactic topical antibiotic should be taken for a few days, especially in high-risk occupation people as farmers, who have an increased risk for trauma through vegetable matter [41].

## Conclusions

According to WHO, 1.9 million people have corneal blindness due to the opacification of the cornea, which accounts for about 5% of the total patients who have blindness. The various conditions which progressively lead to corneal blindness are infectious such as herpes simplex keratitis, bacterial keratitis, fungal keratitis; glaucoma; trachoma; non-infectious uveitis; keratoconus; and dry eye disease. These conditions cause disruption and damage to the structural integrity of the cornea, which eventually leads to blindness and the disturbance of visual acuity.

The management of corneal blindness depends on the precipitating disease that is leading to the blindness. One of the significant clinical modalities that are preferred is corneal transplantation. Hence setting up eye banks all over the country is the need of the hour to decrease the prevalence of corneal blindness. Other than corneal transplantation, conservative therapy is given, like avoiding the risk factors that are leading to the specific disease or opting for modified lifestyle modalities. Topical antibiotics and steroids are also provided for treatment purposes in case of infectious disease.

Some of the recent advancements in the transplantation of cornea are iOCT, FALK, and the bioengineered cornea. The development of the technique of bioengineered cornea is helpful in coping with the increasing demand for cornea. Therefore, to reduce the prevalence of preventable blindness of cornea, proper measures should be taken and health interventions should be implemented globally.
